# Expectation is jointly shaped by global and transitional probabilities

**DOI:** 10.3758/s13423-026-03034-y

**Published:** 2026-11-10

**Authors:** Austin Luor, Frederic Dick, Lori L. Holt

**Affiliations:** 1 Department of Psychology & Center for Perceptual Systems, The University of Texas at Austin, Sarah M. & Charles E Seay Building, 108 E Dean Keeton St., Austin, TX 78712 USA; 2 Department of Experimental Psychology, University College London, London, UK

**Keywords:** Statistical learning, Auditory, Perception, Transitional probability

## Abstract

Humans implicitly extract statistical regularities from sensory input across multiple perceptual dimensions. But it is unclear how statistical learning builds expectations that in turn may affect perceptual decisions, especially in the context of co-occurring regularities. In audition both transitional probabilities (across successive sounds) and global probabilities (sounds’ overall frequency of occurrence) shape perception. Here, we investigated how these two forms of statistical structure jointly influence perceptual judgments. Participants heard a 70-ms cue tone followed by a 50-ms or 90-ms target tone and judged whether the target tone was short or long. Statistical regularities were embedded along a task-irrelevant dimension, tone frequency, allowing for assessment of how implicit statistical learning unfolds when regularities are orthogonal to task goals. A sensory match in cue and target tone frequencies provided no advantage to duration decision response times. However, duration decisions were faster when cue tone frequency predicted target tone frequency across a high transitional probability. Target tones with high global probability drove faster duration decisions compared to those with low global probability. The combined evidence indicates that global target probability and cue–target transitional probability exerted similar magnitude effects, but their combined influence was not additive. Thus, even in active tasks demanding attention to a specific dimension (duration), listeners implicitly learn statistical structure along an irrelevant dimension (frequency) and track multiple regularities. This work advances our understanding of how layered statistical regularities guide prediction in goal-directed listening contexts.

Organisms effortlessly and implicitly detect, extract, and learn patterns of information (Armstrong et al., 2017; Frost et al., 2019; Santolin & Saffran, 2018). In audition, listeners form statistics-driven expectations of what they will hear (Falandays et al., 2021; Sohoglu & Davis, 2020; Theodore & Monto, 2019). They track transitional probabilities across artificial speech syllables (Saffran et al., 1996), patterns of correlated acoustic features (Idemaru & Holt, 2011; Lehet & Holt, 2020), the average spectrum (Holt, 2006; Laing et al., 2012), and evolving vocal regularities (Holmes et al., 2021; Kleinschmidt, 2019; Tzeng et al., 2021; Zhang & Holt, 2018), all of which affect speech recognition.

Humans also learn input regularities that shape word segmentation (Perruchet & Vinter, 1998; Saffran et al., 1996; Thiessen et al., 2013), phonological categories (Maye et al., 2002; Thiessen & Saffran, 2003), and syntactic structures (Thompson & Newport, 2007; Tomasello, 2000). The Competition Model proposes that statistical learning supports language processing by enabling individuals to detect and weight linguistic cues based on input frequency and reliability, thereby resolving competing cues (Bates & MacWhinney, 1989).

Yet although multiple (sometimes competing) regularities are the norm for natural input, studies often examine the influence of a single statistical regularity. For instance, neutralization of global probability reveals an influence of transitional probabilities across syllables (Saffran et al., 1996), visual-spatial sequences (Kirkham et al., 2002), and shape and color sequences (Turk-Browne et al., 2008). Conversely, other studies show that global probabilities affect the efficiency of visual search (Chun & Jiang 1998, 1999; Geng & Behrmann, 2002, 2005) or attention to scene regularities (Chalk et al., 2010; Chun & Jiang, 1998, 1999; Summerfield et al., 2006) by controlling for transitional probabilities. Similarly, high-frequency words are recognized and produced faster than low-frequency words (Goldinger et al., 1989; Howes, 1957; Jescheniak & Levelt, 1994; Marslen-Wilson, 1987; Navarrete et al., 2006) and naming single printed words occurs faster for high-frequency compared with low-frequency words (Spieler & Balota, 2000). In short, when examined in isolation both transitional probability and global probability exert influences on behavior.

We know much less about how multiple statistical regularities interact. To address this, we examine the joint influence of global and transitional probabilities in the simplest, atomic test case: two sinewave tones. Classic psychoacoustic research by Schröger and Wolff (1998) found that perceptual duration decisions were faster for high-probability tone frequencies, even though tone frequency was irrelevant to the task. In other words, global probability influenced decision speed. A separate line of research points to a role for transitional probability in this simple test case. Mondor and Bregman (1994) showed that listeners judged tone duration more quickly when a cue and target tone shared the same frequency, possibly due to high transitional probability or a sensory match between cue and target. The appeal of these active tasks is that regularities are present across task-irrelevant acoustic *frequency* as listeners direct attention to make perceptual decisions about *duration*. This provides an opportunity to examine implicit statistical learning in an active task.

Here, we examined statistical learning across global probability (how often a target tone frequency occurs) and transitional probability (how reliably a cue tone frequency predicts the target tone’s frequency) in isolation, and when the regularities co-occurred. We also considered the role of sensory match between cue and target tone, known to facilitate perception in other domains (Cheadle et al., 2015; Kok et al., 2012; Rungratsameetaweemana et al., 2018; Summerfield & de Lange, 2014; Wyart et al., 2012).

## Experiment 1: Balanced transitional and global probabilities

We first tested whether there was an influence of the sensory match between cue and target tones’ frequencies on the response time to label the target tone as *long* or *short.* This established a sensory baseline from which to evaluate the influence of global and transitional probabilities.

### Methods

#### Participants

We recruited online participants ages 18–35 years who reported normal hearing (Prolific.com) with compensation at a rate of $10/hour. In a pilot study, we collected data from 30 participants, each making duration decisions across 320 trials. We then compared response times for duration decisions in high versus low transitional probability trials (see Experiment 2). This yielded an effect size of 0.71 for the main effect of transitional probability. Accordingly, we targeted 30 participants per group across experiments that manipulated transitional and/or global probability.

In Experiment 1 we recruited a new 30-participant sample and pooled it with the pilot study to increase power to detect a potentially small influence of sensory match (this also aligns Experiment 1 with Experiments 2 and 3, where pooling across groups differing in counterbalancing resulted in 60-participant samples in analyses). Thus, Experiment 1 reports data from a total sample of 60 participants; four of these participants were removed prior to analyses for poor adherence to the protocol (see criteria in Approach to Analyses), resulting in a final sample of 56 listeners (37 women, 19 men; mean age = 23.6 years, *SD* = 4.4 years).

#### Stimuli

**Fig. 1:**
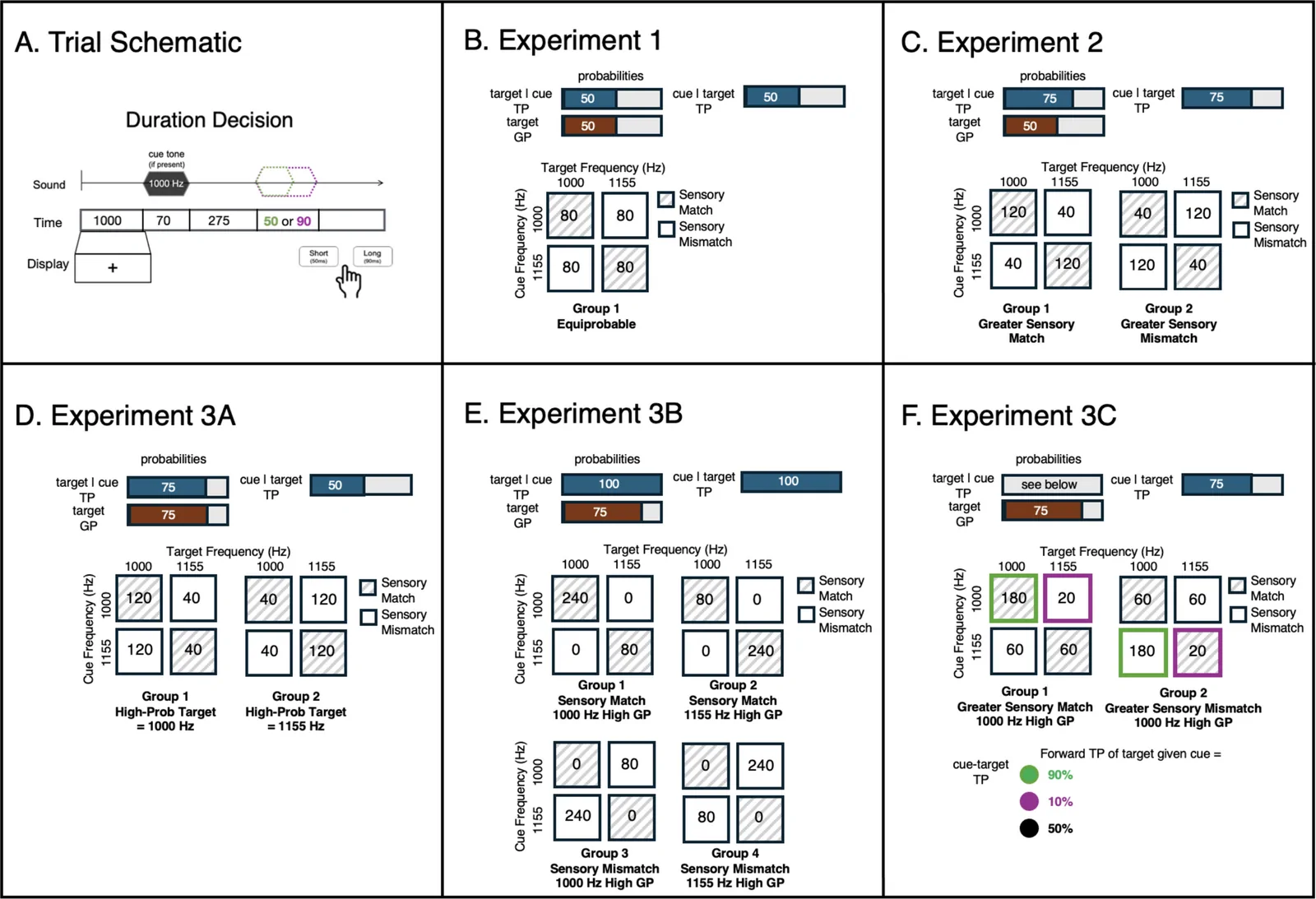
**A. **Duration decision task. Listeners made a duration decision of whether the target tone was short (50 ms) or long (90 ms) in the context of a preceding cue tone (always 70 ms). **B.–F.** For each panel, the colored bars on the top represent the cue–target transitional probability (TP) for both forward (target given cue) and backward (cue given target) relationships as well as target global probability (GP). Below this, a schematic illustrates each of the four trial types according to cue (1000, 1155 Hz) and target (1000, 1155 Hz) tone frequencies. The number inside each cell corresponds to the number of trials of this type in the experiment. Cells with a crosshatch pattern have a sensory match in cue–target frequency; cells without this pattern mismatch. Separate groups of participants experienced statistical regularities counterbalanced across cue and target frequencies and sensory match, as indicated by the labels. In **F**., note that the joint regularities in Experiment 3C resulted in forward transitional probabilities that varied substantially (90:10) across cue–target relationships; this offered an opportunity to test for additive effects

Sinewave tones with 5-ms linear onset/offset ramps were generated digitally and saved in the lossless FLAC format using the Sound eXchange sound processing software (SoX, http://sox.sourceforge.net/) at 44.1 kHz and 16-bit precision. Cue tones were 70 ms, and target tones were 50 ms or 90 ms in duration (see Fig. 1). All tones had a constant frequency of 1000 or 1155 Hz and RMS-matched amplitude.

#### Procedure

We conducted all studies online using Gorilla software (http://gorilla.sc; Anwyl-Irvine et al., 2020). We required participants to use the Chrome internet browser on a laptop or desktop computer (no smartphones or tablets); operating system was not restricted. Before the start of the experiment, a reminder encouraged participants to turn off computer notifications and participants passed a check to assure headphone use; the experiment terminated if participants failed the headphone check across two attempts (Milne et al., 2021).

As shown in Fig. 1A, each trial involved two sinewave tones. The first was always 70 ms and served as the ‘cue’. The second, ‘target’ tone followed 275 ms later and was either 50 or 90 ms, with equal probability and random presentation order. Participants simply judged the duration of the target tone as *long* or *short* with a key press (J or K keys, with the duration-to-key mapping counterbalanced across participants).

Tone frequency was always task irrelevant, and statistically unrelated to tone duration but it carried the statistical regularities that were manipulated across experiments. Before the start of the main task, a block of 32 practice trials included correct/incorrect feedback on duration decisions. The main task commenced after this practice block, with no further feedback. Participants completed the entire protocol in about 40 minutes.

In Experiment 1, each of the four possible cue–target frequency pairings occurred equiprobably (Fig. 1B). This resulted in no reliable transitional probability across cue and target frequencies, and globally equiprobable target tone frequencies. In this statistically neutral context, the cue tone frequency *matched* the target tone frequency on 50% of all trials and *mismatched* on the other 50% of trials. This presented an opportunity to examine whether a sensory match between cue and target frequency influenced duration decision times and established a baseline against which to interpret the manipulations of transitional and global probabilities across cue and target frequencies carried out in Experiments 2 and 3.

#### Approach to analyses

We preprocessed the data using R (R Core Team, 2026; Version 4.6.0) before submitting it to JASP for analysis (Version 0.96, Apple Silicon). From a sample of 60 participants, we excluded four participants who did not exceed at-chance duration decision performance of 54.7%. (Chance was determined based on 10,000 permutations of 320 trials for each participant of all experiments, with an alpha level = 0.05.) There were 19,200 trials among the remaining 56 participants from which we excluded 4,130 trials incorrect responses (21.5% errors). For correct trials, we measured duration decision response time (RT) from the offset of the target tone to the button press, excluding correct responses faster than 300 ms (347 trials, 1.8%) or slower than 1,500 ms (1,331 trials, 6.9%). For all experiments, we performed statistical analyses on raw, unnormalized, RTs. We report statistical values to two decimal places and probability to four decimal places.

### Results

**Fig. 2:**
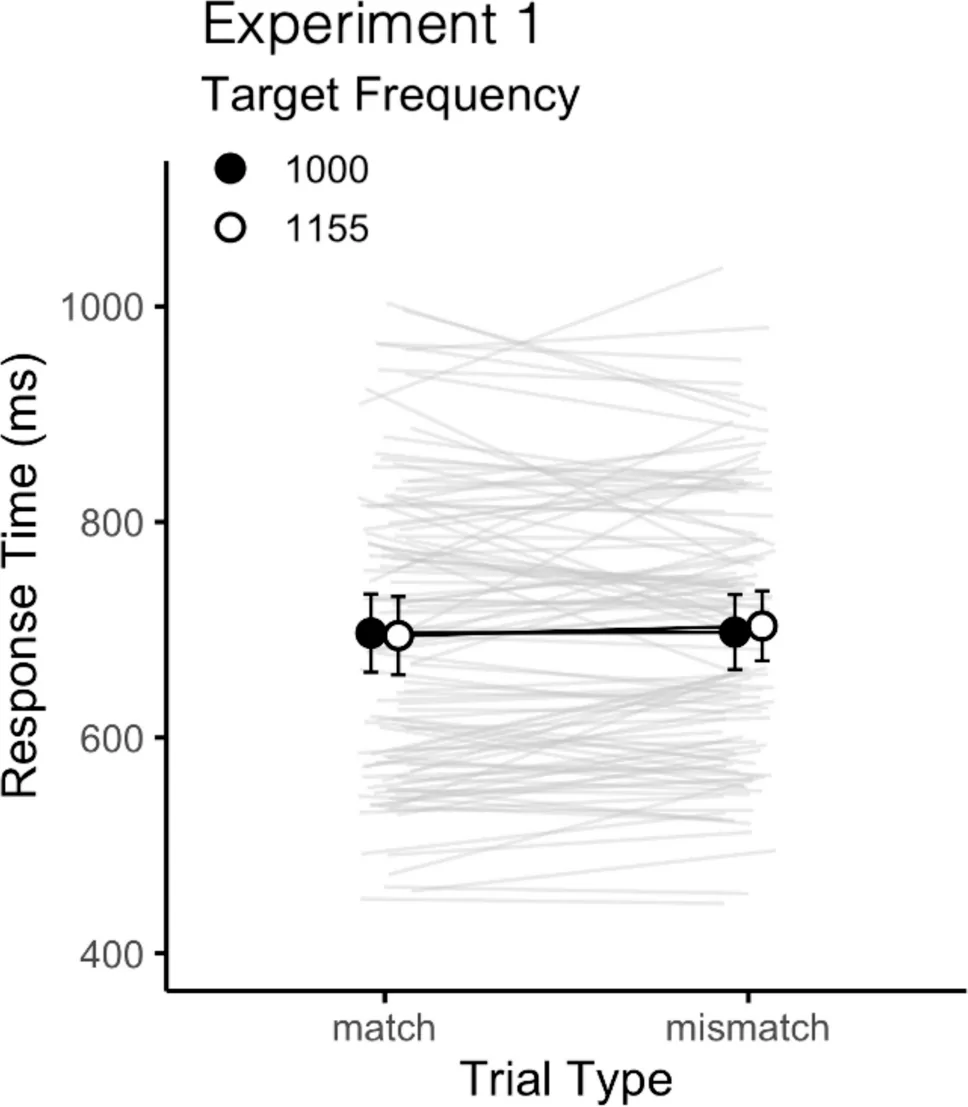
Results of Experiment 1: Balanced transitional and global probabilities. Mean response times to report the duration of the target tone are plotted as a function of whether cue and target tone frequencies matched or mismatched, plotted separately for trials with 1000 Hz and 1155 Hz target tones. The error bars show 95% confidence intervals

Figure 2 shows the results of Experiment 1. A within-subjects ANOVA of target-tone duration decision RTs across cue–target Sensory Match (Match, Mismatch) and Target Frequency (1000 Hz, 1155 Hz) revealed no main effect of Sensory Match, *F*(1,55) = 0.69, *p* = 0.4085, η^2^ = .0058, no main effect of Target Frequency, *F*(1,55) = 0.15, *p* = 0.6980, η^2^ = .0007, and no interaction, *F*(1,55) = 0.72, *p* = 0.4000, η^2^ = .0039. Contrary to evidence from vision where matching sensory features facilitate faster target detection search times (Downing, 1988; Tower-Richardi et al., 2016), we found no evidence that matching acoustic frequency speeds perceptual decision-making about tone duration.

## Experiment 2: Transitional probability, balanced global probability

In Experiment 2, we examined whether task-irrelevant transitional probabilities across cue and target tone frequencies influenced perceptual decisions of tone duration. Prior studies have demonstrated that infants, adults (Saffran et al., 1996, 1999), and even nonhuman animals (Boros et al., 2021; Santolin et al., 2016; Toro & Trobalón, 2005) are sensitive to transitional probabilities across successive events (Santolin & Saffran, 2018). Here, we asked if sensitivity to transitional probability extends to the simple case of auditory tone frequency in an active task in which tone frequency is not task relevant. Specifically, when performing a task directed at an acoustic dimension (duration), we tested whether listeners implicitly track transitional probability across a task-irrelevant dimension (frequency).

### Methods

#### Participants

In all, we tested 60 participants across two groups (see Fig. 1C), with 10 participants excluded for failure to comply with the protocol. The final sample of 50 participants (22 women, 27 men, one nonbinary) had a mean age of 23 years (*SD* = 3.5 years).

### Stimuli

The stimuli were the same as Experiment 1.

#### Procedure

In the context of the task shown in Fig. 1A, we manipulated the relationship of cue and target tone frequencies to create distinct *transitional* (cue-to-target) probability statistics across tone frequency while maintaining equal *global* target frequency probability. Figure 1C illustrates these manipulations. For each of the two groups, the 1000 and 1155 Hz target tones were equiprobable.

The groups differed in the cue–target transitional probability across frequency. In one group, the cue tone frequency predicted the *same* target tone frequency across 75% of trials whereas in the other group, the cue tone frequency predicted a *different* target tone frequency across 75% of trials. Thus, each group experienced the same transitional probability but differed on whether the target tone frequency was predicted by a cue with the same frequency, or a cue with a different frequency. With global probability equated over the target tone frequencies, the forward (target given cue) transitional probabilities were equivalent to the backward (cue given target) transitional probabilities.

It is important to keep in mind that the tone *frequency* was always irrelevant to the duration decision task. Duration, the dimension across which the task was performed, provided no statistical regularity; target tone duration was equiprobable across the experiment. As noted above, in Experiment 2, tone frequencies were each equiprobably 1000 and 1155 Hz so there was no global probability information. In all, the distribution of trial types shown in Fig. 1C established conditions to test whether participants track transitional probabilities across a task-irrelevant dimension.

### Results

**Fig. 3:**
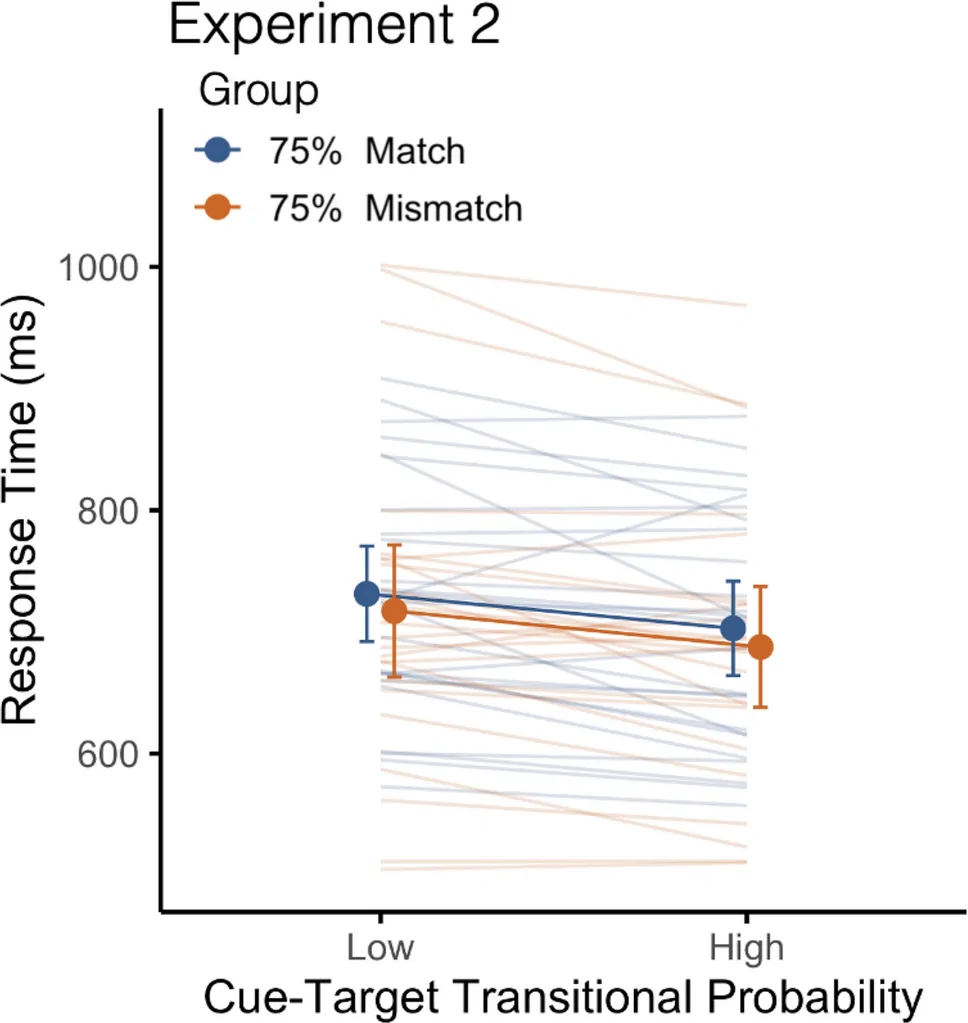
Results of Experiment 2: Transitional probability, balanced global probability. Mean response times to report the duration of the target tone are plotted as a function of low and high cue–target transitional probability, plotted separately for the two groups of participants who differed on whether cue–target frequencies more often matched or mismatched. The error bars show 95% confidence intervals

Figure 3 shows the results of Experiment 2. We analyzed raw response times for correct duration decisions (24% of 19,200 trials) with response times greater than 300 ms (2.6% exclusion; 498 trials) and less than 1500 ms (7.0%, 1,357 trials).

A mixed-model ANOVA with Transitional Probability (Low, High) as a within-subjects factor and Group (Greater Match, Greater Mismatch) as a between-subjects factor revealed a main effect of Transitional Probability, *F*(1,48) = 23.80, *p* = .00001, η^2^ = .0176. Higher transitional probabilities (75%) were associated with faster duration decisions than lower transitional probabilities (25%). In keeping with results from Experiment 1, cue–target match/mismatch did not exert a significant main effect, *F*(1,48) = 0.23, *p* = 0.6341, η^2^ = 0.0045, and there was no interaction with Transitional Probability, *F*(1,48) = 0.01, *p* = 0.9220, η^2^ = 0.0000. In summary, listeners were sensitive to transitional probabilities experienced across the task-irrelevant frequency dimension, such that more predictable target tone frequencies yielded faster duration decisions.

## Experiment 3: Interaction of transitional and global probability

Experiment 3 tested whether task-irrelevant global and transitional probabilities interact in their influence on perceptual decisions (see Fig. 1D–F for schematics). Based on the results of Experiment 2 and given the robust influence of global probability on duration decisions for single tones reported in prior studies (Luthra et al., 2025; Schröger & Wolff, 1998), we anticipated that target tone frequencies with higher global probability would result in faster duration decisions. We also expected an influence of the cue–target transitional probability across frequency, with higher transitional probabilities associated with faster duration decisions.

### Methods

#### Participants

Experiment 3 involved eight separate groups, with a target sample of 30 participants for each group (Fig. 1D–F). To counterbalance the more-probable target frequency across participants, we recruited two separate groups of participants for Experiments 3A and 3C, and four separate groups of participants for Experiment 3B. This resulted in 54 participants in Experiment 3A(six excluded; mean age = 23.2 years; 21 women and 33 men), 55 participants in an Experiment 3B group with matching cue–target frequencies (five excluded; mean age = 24.2 years; 20 women and 35 men), 52 participants in an Experiment 3B group with mismatching frequencies (eight excluded; mean age = 22.2 years; 31 women and 21 men), and 52 participants in Experiment 3C (eight excluded; mean age = 24.8 years; 29 women and 23 men).

#### Stimuli

The stimuli were the same as Experiment 1.

#### Procedure

In the context of the task shown in Fig. 1A we manipulated cue and target tone frequencies in a task-irrelevant manner according to statistical regularities defined as shown in Fig. 1D–F. All Experiment 3 groups experienced one target frequency with a global probability of 75% and the other target frequency with a 25% probability. Assignment of tone frequency (1000, 1155 Hz) as high probability was counterbalanced across groups in Experiments 3A and 3B. In Experiment 3C, 1000 Hz was the high probability target tone frequency across each of the two groups.

With global target frequency probability set at 75:25 across all of Experiment 3, transitional probability manipulations differentiated Experiments 3A–3C, as shown in Fig. 1D–F. In Experiment 3A, we paired cue and target frequencies such that the forward (target given cue) transitional probability was 75:25 across pairs; backward transitional probabilities (cue given target) were equivalent (50:50; Fig. 1D).

In Experiment 3B, the cue tone frequency perfectly predicted the target tone frequency (100%, for both forward and backward transitional probabilities). For each global target frequency probability (1000 Hz 75% probable or 1155 Hz 75% probable), one-half of participants experienced cue–target pairs that always matched in frequency and the other group experienced only mismatching pairs. In all, 4 separate groups participated in Experiment 3B (Fig. 1E).

In Experiment 3C, forward transitional probability and global target probability statistics differentially predicted target tone frequency. For example, in the Sensory Match group, a 1000 Hz cue predicted a 1000 Hz target tone on 90% of trials (see Fig. 1F). By contrast, the 1155 Hz cue was not predictive of target frequency because 1000 Hz and 1155 Hz target tones occurred equiprobably following an 1155 Hz cue tone. The trial structure of the Sensory Mismatch group was the opposite: a 1000 Hz cue tone was equally likely to be followed by a target of either frequency, but a 1155 Hz cue tone was followed by a 1000 Hz target tone on 90% of trials. Thus, if listeners track and use both global probability and forward transitional probability across a task-irrelevant dimension, decision response times should be faster for the cue–target pairs where transitional probabilities are 90:10 and global probability is *also* weighted to the same target frequency (75:25).

### Results

We again analyzed raw response times for correct duration decisions (excluding 19.6%, 18.9%, 20.9% of trials for Experiments 3A, 3B, and 3C, respectively). Among correct trials, we excluded trials with response times less than 300 ms (excluding 2.6%, 4.7%, 2.5%, for Experiments 3A, 3B, 3C, respectively) and trials with response times greater than 1500 ms (excluding 6.4%, 5.6%, 7.1%, for Experiments 3A, 3B, 3C, respectively).

#### Experiment 3A: 75:25 ratio global target frequency probability, 75:25 ratio forward (target given cue) transitional probability, 50:50 ratio backward (cue given target) transitional probability, cue and target frequencies can match or mismatch

**Fig. 4:**
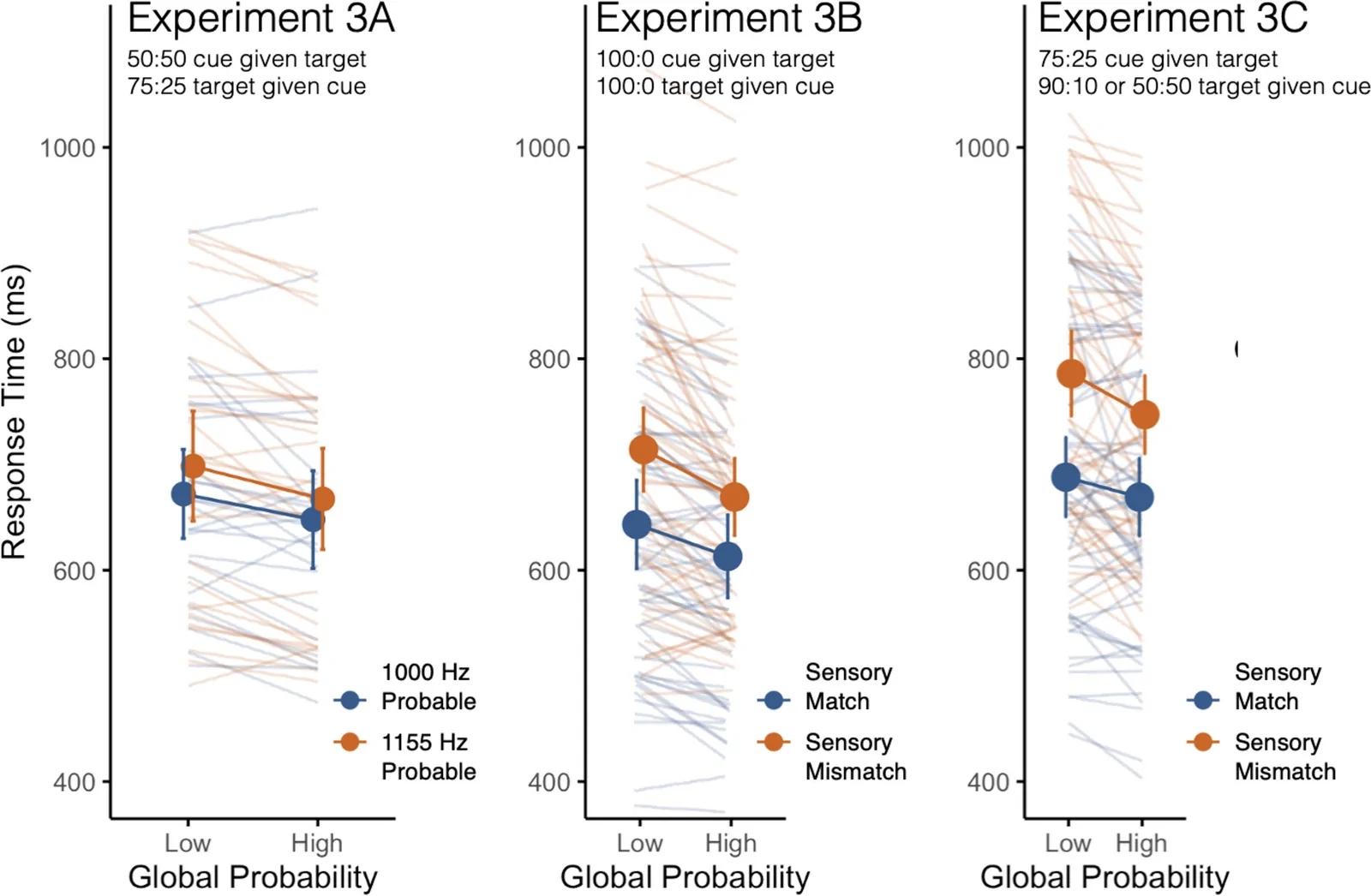
Results of Experiment 3: Interaction of transitional and global probability. Mean response times to report the duration of the target tone are plotted as a function of low (25%) and high (75%) global target probability, which was constant across experiments. Line color in Experiment 3A is plotted according by groups that varied in whether 1000 Hz or 1155 Hz was the high probability target tone. (The within-group manipulation of cue–target frequency match is not plotted). Line color in Experiments 3B and 3C is plotted according to whether the participant group experienced cue–target frequencies that were *more likely* to match or mismatch (100% match or 100% mismatch in 3B, 75% or 25% match in 3C). The error bars show 95% confidence intervals

Based on prior studies (Luthra et al., 2025; Schröger & Wolff, 1998), we predicted that duration decision response times would be faster for higher-probability target tones than lower-probability target tones. We tested this with a mixed-model ANOVA with Global Probability (high, low) and cue–target Sensory Match (match, mismatch) as within-subjects factors, and Group (1000 Hz target more likely, 1155 Hz target more likely) as a between-subjects factor. There was a significant main effect of Global Probability, *F*(1,52) = 26.71, *p* < .00001, η^2^ = 0.0128, such that duration decision response times were faster for target tone frequencies with high global probability than low global probability (see Fig. 4, left panel). There was no effect of Group according to which target frequency (1000 Hz or 1155 Hz) was higher probability, *F*(1,52) = 0.49, *p* = 0.4858, η^2^ = 0.0084, and no interaction between Global Probability and Group, *F*(1,52) = 0.4541, *p* = 0.5034, η^2^ = 0.0002.

Like the results of Experiment 2, we found neither a significant effect of cue–target Sensory Match on participants’ decision RTs, *F*(1,52) = 0.02, *p* = 0.5034, η^2^ < 0.0001, an interaction with Group, *F*(1,52) = 0.12, *p* = 0.7307, η^2^ < 0.0001, nor a three-way interaction among independent variables, *F*(1,52) = 0.39, *p* = 0.5366, η^2^ = 0.0002.

It is important to highlight that global target frequency probability in Experiment 3A is also conveyed by *cue* frequency. To maintain an equiprobable (50%) backward (cue given target) transitional probability with a global probability difference in target frequency (75:25), the forward transitional probability for *both* cue frequencies must also be in a 75:25 ratio. In other words, while cue tone frequency does not provide *differential* information about target frequency, both cue frequencies have higher forward (target given cue) transitional probabilities for the globally more probable target frequency. Thus, as an additional baseline comparison case where there is *only* global probability information about target frequency, we compared Experiment 3A with a previously published study identical to Experiment 3A except that no cue was presented before the target tone (Luthra et al., 2025; Experiment 4A).

For this analysis we calculated the RT difference between high and low global probability target tones for each experiment and compared the high versus low global probability difference scores across the two experiments with a Welch’s ANOVA. This revealed no significant difference in response time facilitation to high versus low global probability targets when preceded by a generally predictive cue tone (the present Experiment 3A) compared with only a target tone with no preceding cue tone (Experiment 4A of Luthra et al., 2025), *F*(1,110.72) = 0.0046, *p* = 0.9462, *d* = 0.0127. This between-participants comparison suggests that the facilitation of duration decision response times in Experiment 3A was driven by the global probability of the target tone, without further meaningful facilitation by the forward transitional probability provided by the cue frequency.

In summary, Experiment 3A revealed that listeners’ duration decisions were faster for tones whose carrier frequency was more probable—an effect of global probability—and that this global probability effect was not facilitated by the presence of a cue tone with higher forward transitional probability. As with Experiment 2, RTs were not significantly affected by whether the cue and target tone matched in frequency.

#### Experiment 3B: 75:25 ratio global target frequency probability, 100:0 ratio forward and backward cue–target transitional probability

In Experiment 3B, the Global Probability of target tone frequency was 75:25 for all participants, like Experiment 3A (see Fig. 1E for full design schematic). However, in 3B, cue tone frequency perfectly predicted target tone frequency (transitional probability = 100%), unlike Experiment 3A. Also, in contrast to Experiments 1 and 3A, assignment of the high-probability Target Frequency (1000, 1155 Hz) and cue–target Sensory Match (versus mismatch) were between-subject factors.

A mixed-model ANOVA showed that response times to high compared with low Global Probability targets were significantly faster, *F*(1,103) = 76.26, *p* < 0.0001, η^2^ = 0.0164 (see Fig. 4, middle panel). This facilitation by higher Global Probability was not significantly modulated by Target Frequency, *F*(1,103) = 3.08, *p* = 0.0822, η^2^ = 0.0007. As with Experiment 3A, we asked whether the overall global probability effect on RTs was enhanced by the presence of a 100% predictive cue tone by comparing the difference between RTs to low and high probability target frequencies in Experiment 3B to those of Experiment 4A of Luthra et al. (2025) where listeners heard a target tone only. A Welch’s ANOVA did not show a significant difference in probability effects on RT between the 75:25 global probability and 100:0 transitional probability of Experiment 3B and the no-cue 75:25 global probability difference in Luthra et al. (2025), *F*(1,136.95) = 1.76, *p* = 0.1865, *d* = 0.2099.

Unexpectedly given the results of Experiments 1 and 3A, there was a significant main effect of cue–target Sensory Match, *F*(1,103) = 5.32, *p* = 0.0231, η^2^ = 0.0461. Durations decisions were slower overall among participants who experienced cue–target sensory mismatches (e.g., 1000 Hz cue, 1155 Hz target), compared with those who experienced matching cue–target frequencies. In line with the findings from Experiment 1, there was no significant main effect of which Target Frequency was higher probability, *F*(1,103) = 0.51, *p* = 0.4789, η^2^ = 0.0044, and no significant interaction with cue–target Sensory Match, *F*(1,103) = 1.92, *p* = 0.1694, η^2^ = 0.0166.

There was also a significant three-way interaction of all factors, *F*(1,103) = 5.77, *p* = 0.0181, η^2^ = 0.0012, albeit of very small effect size. This was due to a stronger global probability effect among the group of participants for whom cue–target frequencies always mismatched and 1155 Hz was the probable target frequency.

In summary, Experiment 3B showed that higher global target probability drove faster RTs. The addition of a deterministic frequency cue did not significantly augment this facilitative effect, compared with participants from Luthra et al. (2025) who experienced a difference in global target probability alone. In contrast to the lack of within-subjects cue–target frequency match effects in Experiments 1 and 3a, the participants who experienced only cue–target frequency mismatches had overall higher RTs compared with those who experienced only matching cue–target frequencies.

#### Experiment 3C: 75:25 ratio global target frequency probability, 90:10 or 50:50 ratio forward transitional probability, 75:25 ratio backward transitional probability

The design of Experiment 3C allowed us to ask three different questions regarding the effects of task-irrelevant global and transitional probability on decision reaction times. We first addressed the overall question of how RTs are affected by Global Probability and proportion of cue–target Sensory Match using a mixed-effects ANOVA (as in Experiment 3A and 3B). Because the experimental design was not fully factorial, we tested specific hypotheses about the separate and combined influence of global and transitional probability via targeted independent-samples *t* tests on the difference scores between RTs to low- versus high- global or transitional probability trials.

The mixed-model ANOVA with Global Probability (low, high) of the target frequency as a within-subjects factor and proportion of Sensory Match (low, high) as a between-subjects factor showed a main effect of Global Probability, with significantly slower RTs to low- versus high-probability target frequencies, *F*(1,50) = 36.80,* p* < 0.00001, η^2^ = 0.0110 (see Fig. 4, right panel for this and next results).

Similar to Experiment 3B, there was a main effect of Sensory Match such that the participant group experiencing a majority of mismatched cue–target frequencies had significantly slower RTs than those in the group with majority sensory-matched cue–target frequencies, *F*(1,50) = 5.88, *p* = 0.0190, η^2^ = 0.1024. There was also a small-effect interaction of Global Probability of the target frequency with the Sensory Match probability, *F*(1,50) = 4.35, *p* = 0.0421, η^2^ = 0.0013. An independent Welch’s* t* test comparing the two groups’ difference between RTs across the Global Probability of target frequencies found that participants who experienced more cue–target sensory mismatches showed a larger global target frequency probability effect compared with those experiencing predominantly matching cue–target frequencies, *F*(49.99) = 4.37, *p* = 0.0416, *g* = 0.5710.

In all experimental results thus far, higher global target frequency probability drives faster decision reaction times. The design of Experiment 3C allows us to ask whether *global* target probability differences accrued over all trials of an experiment still exert this effect even in trials with a conflicting statistic conveyed by the *transitional* probability of cue–target frequency. To do this, we compared RTs to targets with experiment-wise 75:25 global target frequency probability ratio but only included trials with 50:50 forward transitional probability (in the Fig. 1F schematic, the pair of cells with 60 trials each). Based on the global probability effects we observed in Luthra et al. (2025) and current Experiments 3A and 3B, we predicted faster decisions for the subset of trials with targets whose frequency was more probable across the entire experiment, even when forward transitional cue–target probability was equal (black boxes in Fig. 1F).

In contrast to our prediction, a single-sample t-test revealed no significant difference in response time for high versus low Global Probability over these transitional-probability-matched conditions, *t*(51) = 0.66, *p* = 0.5134, *d* = 0.0913. In other words, equating cue–target transitional probability appeared to cancel out the influence of experiment-wide Global Probability differences on decision times.

**Fig. 5:**
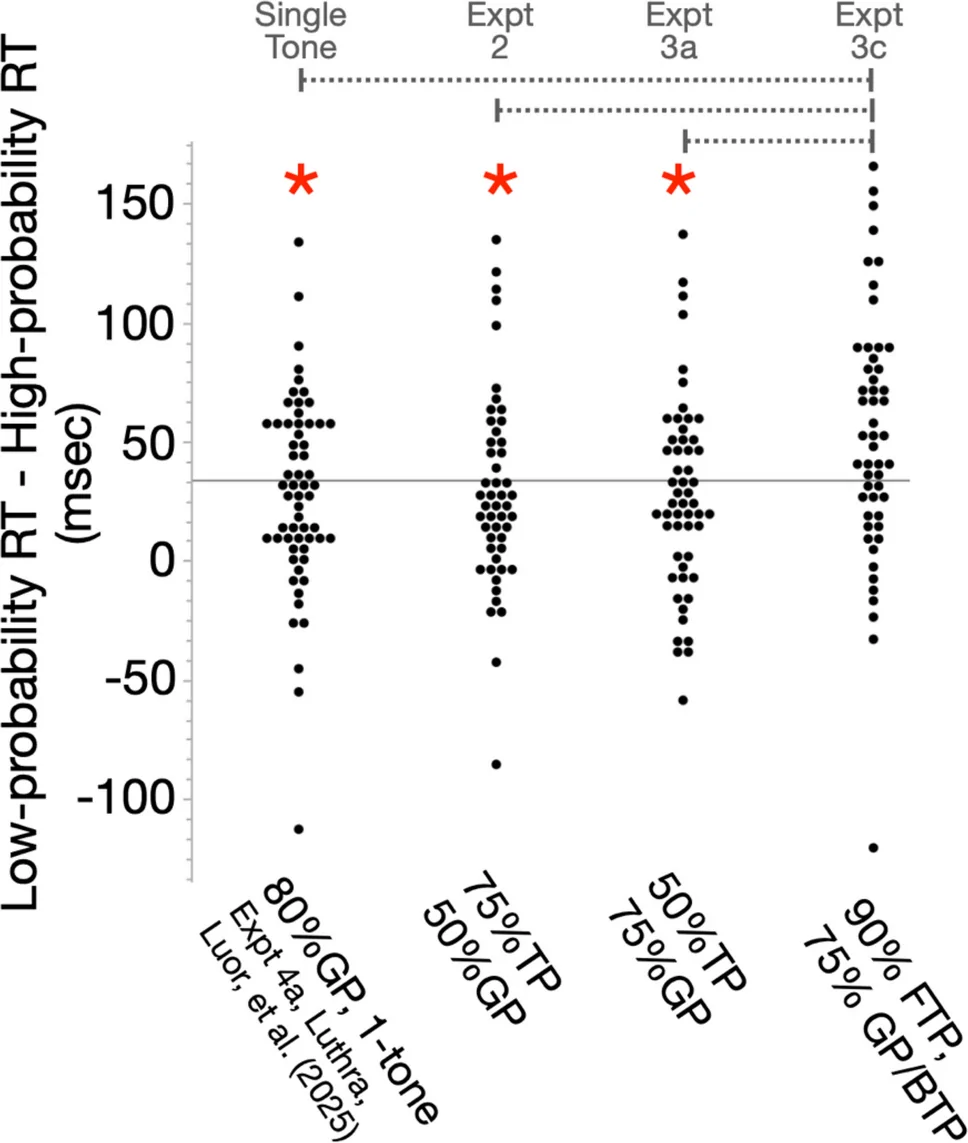
Magnitude of the 90:10 transitional probability/75:25 global probability effect from Experiment 3C compared with global probability effects from Experiment 4A of Luthra et al. (2025) and present Experiment 2 and Experiment 3A. Each point shows a single participant’s difference in reaction times (RTs) for low minus high probability target tones as evoked by a) global target tone probability (GP); b) cue–target forward and backward transitional probability (TP); c) forward or backward transitional probability (FTP, BTP). Percent refers to the higher probability event. The asterisks indicate that RT differences in the condition were significantly smaller than the transitional + global probability RT effect from Experiment 3C, as tested using Steel’s method for multiple comparisons against a control value

Finally, we asked whether a highly skewed cue–target frequency transitional probability (90:10) that is congruent rather than conflicting with global target frequency statistics (75:25) would further *augment* probability effects on duration decision RTs, relative to 75:25 global target probability alone, or 75:25 transitional probability alone. To test this hypothesis, we computed the RT difference between low and high transitional + global probability trials in Experiment 3C (in the Fig. 1F schematic, RTs for trials outlined in magenta minus those outlined in green), and compared the magnitude of this RT difference with that observed for low minus high global target frequency probability trials in Experiment 2 and 3A, as well as the single-tone Experiment 4A of Luthra et al. (2025). Subject-wise data for each experiment are shown in Fig. 5. Nonparametric comparisons (corrected for multiple contrasts using the Steel method) showed that the RT effect evoked by the combination of high (90:10) cue–target transitional and global (75:25) were significantly larger than those driven by: (a) 75:25 global probability of a single tone frequency in Experiment 4A of Luthra et al. (2025), *z* = 2.65, *p* = 0.0219; (b) 75:25 cue–target transitional probability + 50:50 global probability in Experiment 2, *z* = 2.68, *p* = 0.0201; and (c) 75:25 global target probability with 50% transitional probability in Experiment 3A, *z* = 2.76, *p* = 0.0160.

As well as replicating the Experiment 3B group effect of slower overall reaction times to mismatching cue–target frequencies, Experiment 3C showed interacting effects of global target and transitional cue–target probability on duration decision times. On the one hand, when specific cue–target frequency combinations were associated with equal transitional probabilities, participants did not show significant differences in reaction times to target frequencies that differed in global probability across all experiment trials. On the other hand, in trials where cue–target transitional probabilities were highly asymmetric (90:10) *and* the 75:25 global target frequency probabilities were aligned with transitional probabilities, then the same participants showed larger probability effects compared with RT differences in other participant groups who experienced either global or transitional asymmetric probabilities only.

## General discussion

The environment is layered with multiple, overlapping regularities. But which do learners prioritize when multiple, regularities evolve simultaneously across input? Here, we found that task-irrelevant statistical regularities influence task-relevant perceptual decisions. Specifically, the transitional probability of acoustic frequency across two tones affects duration judgments: listeners responded faster when a preceding cue tone frequency predicted the subsequent target tone frequency. Likewise, the global probability of the frequency of the target tone upon which duration decisions were made exerted a similar-magnitude influence. Decisions were faster for tones with higher-probability frequencies. This effect persisted whether cue and target matched or mismatched in frequency. Moreover, the influence of statistical learning emerged implicitly in an active task demanding attention to a different auditory dimension, duration. Transitional probability (cue–target) and global (target only) probabilities also interacted: when cue–target transitional probability was equivalent, listeners reaction times were not significantly affected by the experiment-wide biased probability of the target frequency. This contrasts with the strong effects of global probability observed in the other conditions, and in previous experiments and different tasks. However, when participants experienced strong combined transitional and global probability distributions that converged rather than competed, participants showed even larger reaction time differences when making duration decisions tones with probable and improbable frequencies and cue–target frequency combinations. Future experiments will be needed to understand how relative weighting of transitional and global probability extends to other, less sensorily and statistically impoverished contexts.

When participants experienced both sensory matches and mismatches between cue and target frequencies *within an experiment* (Experiments 1, 2, and 3A), we consistently observed no evidence for facilitation of duration decisions by a sensory match. This is unexpected, given prior research in auditory attention showing that listeners are generally better at detecting signals when they occur within an expected-frequency range (Botte, 1995; Dai & Buus, 1991; Dai et al., 1991; Greenberg, 1969; Greenberg & Larkin, 1968; Penner, 1972; Scharf et al., 1987). This might be accounted for by inhibition of return (IOR) effects whereby responses to sensory-matched frequencies are slower at longer stimulus onset asynchronies (e.g., 750 ms) compared with shorter asynchronies (e.g., 150 ms) for which there can be facilitation. This is thought to arise from an attentional mechanism that inhibits returning to a previously cued frequency that exhibits a longer latency (Mondor et al., 1999). In our task, the 275-ms SOA targeted middle-ground along the IOR curve, potentially contributing to our consistent observation of a lack of facilitation for sensory-matched cue–target pairs.

However, we did observe faster reaction times for *participant groups* in Experiments 3B and 3C who experienced only mismatching (3B), or predominantly mismatching (3C) cue–target frequencies, compared with participants in the same experiment experiencing all or predominantly matching cue–target frequencies. Particularly given the consistent lack of such effects within-subjects in all other experiments (where we were well-powered to detect such differences), these were unexpected findings, and particularly challenging to interpret unambiguously, given potential cohort differences in overall reaction time. Follow-up studies to investigate these effects will require an additional motor response baseline to rule out underlying group differences in processing speed and attentiveness.

Moving to organisms’ sensitivity to transitional probability information, our results extend well-documented influences of transitional probabilities on behavior (Santolin & Saffran, 2018) to an *active* task. Whereas many studies have focused on passive statistical learning paradigms (Fiser & Aslin, 2002; Mc Mullen & Saffran, 2004; Romberg & Saffran, 2010; Turk-Browne, 2012; Zhao et al., 2013) statistical regularities are learned implicitly across active tasks (e.g., Anderson, 2026; Hodson et al., 2023; Luthra et al., 2025; Roark et al., 2022), as well. Our task builds on this evidence, inspired by early probe-signal studies (Greenberg & Larkin, 1968; Mondor & Bregman, 1994), to show that statistical learning leaves its mark on the efficiency of perceptual decision making even in contexts in which statistically structured input is orthogonal to the goals of an active task.

It might seem inefficient for a system to track regularities irrelevant to the task at hand. However, in natural environments with shifting regularities and task demands it may be effective to ‘keep an ear out’ by tracking evolving regularities that have potential utility for future behavior (Frost et al., 2025). Schröger and Wolff (1998), who pioneered the duration-decision task we use here, argued for a memory-based mechanism that detects deviance from expectations. This orients attention to unexpected (low global probability) tones leaving fewer resources for duration decisions and slowing response times to tones with lower-probability frequencies. Mondor and Bregman (1994), in a case of convergent experimental design, used a very similar duration-decision paradigm. They concluded that the influence of global probability arises from selective attention to the expected (high global probability) tones such that decision making is facilitated. These opposing perspectives highlight the interpretational challenges that exist in literatures on expectation driven by statistical learning and the intersection of this learning with attention (Press et al., 2020) that may be illuminated in studying finer-grained distributional manipulation of global probability (see Luthra et al., 2025).

In this context, studies of the influence of joint statistical regularities in the simple ‘atomic’ case of tone frequencies that we have studied here are an important tool in revealing what the system brings to learning across more complex and jointly co-evolving, dynamic statistics typical of natural input, like speech. As an example, Frank et al. (2013) had adults passively listen to a large corpus of synthesized speech over approximately an hour a day for 10 days. This input was composed of 1,000 different words carrying a Zipfian frequency distribution such that some carried high global probability, but most occurred infrequently. Words were concatenated randomly (but without immediate repetitions) such that there were higher transitional probabilities within, compared with between, words. In the context of these joint regularities, participants learned to segment the novel words and retained this performance across 1–2 months. Retention for words with the highest *global* probability persisted even three years later. Thus, statistical learning scales to complex input carrying multiple regularities (see also Kurumada et al., 2013). Conducted in conversation with studies of more complex input, studies that exploit the fine control over regularities afforded by simpler regularities like those used here, can establish the conditions under which multiple sources of prediction influence behavior.

The present results also highlight the importance of studying statistical learning in the context of multiple regularities: in Experiment 3C, global and forward transitional probabilities interacted additively. In the simple, atomic paradigm of the present study, it was also possible to observe a more subtle pattern of interaction that emerged across specific cue–target pairs. Whereas the Experiment 3C session-wide forward transitional probability was 75:25, the simultaneous manipulation of target global probability rendered some cue–target pairs balanced (50:50) and others skewed (90:10) in their forward transitional probabilities. Participants were sensitive to this difference, with the reliability of information conveyed by specific cue–target pairs affecting duration decision response times. Specifically, duration decision response times were facilitated above and beyond the influence of global probability alone for skewed cue–target pairs. But, for balanced cue–target pairs, there was no influence of global probability. This is curious in the context of the significant influence of target global probability in the context of balanced transitional probabilities in Experiment 3A. These differences would seem to arise from listener sensitivity to the mixed relative reliability of specific cue–target pairs in Experiment 3C. Linking again to more natural listening contexts like speech, the reliability differences arising from interactions across statistical regularities align well with theoretical accounts of cue competition whereby listeners assign relative weights to acoustic dimensions based on diagnosticity (Holt & Lotto, 2006), adjust weights based on reliability (Idemaru & Holt, 2011), and affect perceptual processing at multiple levels (Lehet & Holt, 2020). Also motivated by consideration of statistical learning across speech, it may be productive for future studies to examine the ‘bigram’ probability of the cue–target pairs, especially as a function of time as longer exposure to high cue–target transitional probability drive learning.

In summary, the present studies demonstrate that listeners track transitional and global probabilities across stimulus dimensions that are not directly relevant to the task at hand, and this learning impacts the speed of perceptual decision making. Listeners are sensitive to both global and transitional probability when other input regularity is held constant, with each exerting a similar magnitude of influence. We also saw initial evidence that listeners differentially utilized these sources of information when both were available, such that experiment-wide unequal global probability did not significantly affect decisions when transitional probabilities were equivalent. In all, the present studies highlight that statistical learning across multiple regularities proceeds even across a task-irrelevant dimension with an impact on the speed of perceptual decision making in an active task.

## Data Availability

Data and materials can be found online (https://osf.io/mw5z3/).
